# The Contribution of Community Health Education to Sustainable Control of the Neglected Zoonotic Diseases

**DOI:** 10.3389/fpubh.2021.729973

**Published:** 2021-10-12

**Authors:** Caitlin Butala, Jenna Fyfe, Susan Christina Welburn

**Affiliations:** ^1^Zhejiang University School of Medicine, Zhejiang University-University of Edinburgh Institute, Zhejiang University, Haining, China; ^2^Infection Medicine, Deanery of Biomedical Sciences, Edinburgh Medical School, College of Medicine & Veterinary Medicine, The University of Edinburgh, Scotland, United Kingdom

**Keywords:** one health, neglected zoonoses, health education & awareness, the vicious worm, Meena Communication Initiative

## Abstract

Effective and sustainable control of the Neglected Zoonoses (NZDs) demands a One Health approach. NZDs largely impact on individuals in low- and middle-income countries, disproportionally affecting resource poor communities with poor access to veterinary and human health services and to clean water and which are intrinsically dependent on animals for their livelihoods. Many NZDs in humans can be treated, but treatment is often complex and expensive. Similarly, while tools for prevention of transmission may exist, they are complex and expensive to adopt at the scale required to be effective. The cost of intervention for NZDs is high when compared to the public health benefits alone, but costs are easily outweighed by full cross sector analysis and when monetary and non-monetary benefits to all stakeholders are considered. Education is a key tool, often overlooked in favor of more complex solutions for the control of NZDs. Successful education programs have been targeted to children of school age for *Taenia solium* in Kenya, schistosomiasis in Nigeria, and soil transmitted helminths in China. A Snakes and Ladders board game, designed to teach children about schistosomiasis and encourage compliance with mass deworming programs, deployed in Nigerian schools, showed a 67% increase in knowledge of praziquantel and 65% of children who had previously rejected treatment requested the drug at school. For soil transmitted helminths in China, presentation of health information in cartoon format rather than in poster format, showed post-assessment knowledge to be 90% higher. With the rise in affordable smart-phone technology, internet access and airtime in communities in low- and middle- income countries e-education is an increasingly attractive proposition as an intervention tool for the NZDs. The Vicious Worm, a computer based educational health tool that has been designed around the prevention of *Taenia Solium* has shown remarkable efficacy in affected communities in which it has been deployed with participants applying the principles learned in their communities. This review explores the successes and benefits of education as a control tool for the NZDs.

## Introduction

Zoonotic diseases, diseases that transmit between animals and humans, cause morbidity, mortality, and loss of productivity in both groups which can greatly impact the global burden of a disease. Of Emerging Infectious Diseases (EID), zoonotic diseases comprised 60.3% of all EIDs between 1940 and 2004, a significant number that has not decreased in the last 15 years (1). While some Zoonotic diseases are in the forefront of the public's eye, for example, Covid-19 and Ebola, a majority of zoonoses are neglected. Neglected Zoonotic Diseases (NZD) make up the bulk of Neglected Tropical Diseases (NTD), which are predominantly endemic in Low- Middle Income Countries (LMIC), particularly in areas where people live in close proximity with their livestock. These subsistence farmers are amongst the poorest people globally, part of the so called “bottom billion”, who bear a significant proportion of the NTD burden (2). A significant proportion of this burden is made up of soil transmitted helminths (STH), an estimated 1.5 billion people are thought to be infected with one of the STHs (3). A significant part of the NTD elimination strategies have focused on deworming programs. One Health is an interdisciplinary approach to health, not only of humans but animals and the environment as well. It is the driving force behind many current health policies around the globe and is instrumental in the long-term eradication goals of NTDs and particularly NZDs (4–6). It is essential for reaching the World Health Organization's (WHO) target goals of elimination for NZDs, as well as prevention for potential future EIDs.

### History of Worms and Deworming Programs

Parasitic worms have been a part of human history since time immemorial, with the first direct evidence from 8000 BC, but it is suspected that parasitic worms have been in humans for 50 thousand years or more (7). Parasitic worms have been found in Ancient Roman latrines, in Egyptian mummies, and Ancient Chinese burials and are still found around the world in modern humans (8–11). There are 277 species of helminths and 66 species of protozoa where humans act as the main host or an incidental host (12). In modern times, only 90 of these species are common occurrences (13). One of the most well-known subsets of NTDs are the worm-based diseases, including STH, schistosomiasis, cysticercosis/neurocysticercosis and dracunculiasis.

Deworming, particularly in school aged children (SAC) population, became a popular cause after the seminal study by Miguel and Kremer in 2004. Their research stated that deworming children in Kenya with biannual treatments of albendazole and annual treatments of praziquantel, not only significantly decreased school absenteeism at the treated schools but there was also a reduction of school absenteeism at schools in up to a 6 km radius. The authors acknowledged that despite the reduction in absenteeism, the deworming program did not provide “any evidence that deworming increased academic test scores” (14). This study became the rallying cry for many advocates of deworming and has been touted by non-governmental organizations, politicians, researchers, and many others as the justification for the time, effort, and money spent on deworming programs around the world.

In 2012, following the London Declaration on Neglected tropical Diseases, pharmaceutical companies GlaxoSmithKline (GSK) and Johnson& Johnson announced their plan to donate 600 million of doses of antihelminthic drugs annually to endemic countries through to 2020. Charities and nonprofit organizations donated an additional 100 million doses each year (15). These donations have enabled countries to increase their control of parasitic worms through existing governmental programs (16). As a result of these donations many countries implemented or expanded their in-school deworming programs (16–18). In these programs SAC are given annual or biannual doses of anti-parasitic drugs distributed by teachers or community health workers at the schools. Over the last 18 years, GSK has donated 8.5 billion doses of albendazole and has created a manufacturing plant in India for the sole purpose of making albendazole (19). The London Declaration created a framework on how to move forward to achieve the goals of eliminating these diseases by 2020. Because of the declaration and the emphasis on elimination, deworming programs received increased attention and money from both governments and non-governmental organizations.

Efforts have been made to eliminate worms under the premise that it would improve health, school performance, and national Gross Domestic Production (GDP). In an inventory performed in 2010, there were 120 Non-governmental Organizations (NGO) that work to eliminate worms and provide anti parasitic drugs and education to communities (20). These groups are in addition to government intervention programs run in endemic countries, and in most locations the two sections work together to improve intervention success.

Since the initial study by Miquel and Kremer in 2004, there have been attempts to replicate their results to varying degrees of success. There is much debate in the biomedical sciences community as to whether deworming actually creates the benefits that are touted (21–27). While there are policy makers and politicians who cite increases in school attendance, weight gain, reversing anemia as the major benefits of deworming in school-based Mass Drug Administration (MDA) programs, the results are not so clear cut. Taylor–Robinson et al. have conducted a systematic review for the Cochrane Database of Systematic Review, with several updates, on the subject and have consistently found that there is no significant evidence of deworming having an effect on weight gain or hemoglobin, no reliable evidence on school performance, and evidence for improvements in school attendance are limited (28, 29). In their research for the systematic reviews, they found errors in calculations or with cluster randomized designs in several experiments related to deworming SAC, few of which have been addressed or corrected by the authors of those studies.

Miguel and Kremer remain steadfast in their results that deworming contributed to a reduction in school absenteeism and have published a 20 year follow up on their original study. In this study they highlight a continued impact in the lives of those subjects, suggesting that children who received albendazole treatments have a 13% increase in hourly wages and are 9% more likely to live in urban areas (30).

It is clear that more research and reliable replication studies are needed to define and determine the effects of deworming on SAC, as these deworming programs continue to be the main intervention for elimination of STH.

### Health Education

Health education is an important part of disease control, although it is often mentioned as an afterthought, a nonspecific intervention compared to the massive MDA intervention programs. There is limited research on the efficacy and impact of health education as a direct intervention, there is a need to study the effects that education alone can have on NTDS as “education itself is a health intervention” (31).

Health education can take many forms and has been included in interventions around the world. Historically, education to the community came in the form of pamphlets, training a community health worker to explain to the village communities, and radio or tv adverts. Posters are a traditional method for disbursing information on health topics to school aged children; however in an ever-increasing digital age that allows for interaction, posters often fall short on engaging children. While students may look at the posters, and posters are better than no information at all, there are more effective tools today to present health education. Over the last 20 years there have been multiple forays into health education via cartoons, films, radio programs, comic books, and cultural performances. In more recent years it has expanded to mobile apps and texts from Ministry of Health departments with the growth of mobile phone usage around the world (32).

Mobile networks are available for almost 95% of the global population, and mobile broadband networks that are at least 3G or higher reach 84% of the global population, but only 67% of the rural population (33). In LMIC access to internet available devices may be limited and data networks can vary in both quality and cost, there is continually ongoing development in many countries to reduce these issues. Google and several telecommunication companies have been experimenting with internet balloons and drones that would provide internet in areas with limited or no internet coverage, however to date these have been unsuccessful (34–38). In the future this could allow students more access to health education in areas with prohibitively expensive data networks.

## ASIA

### Meena Communication Initiative

The Meena Communication Initiative (MCI) was a groundbreaking Education Entertainment (EE) project created in 1991 as a human rights intervention campaign focused on girls rights in Southeast Asia. It was funded by UNICEF and developed with input from 10 countries to create a main character, Meena, who could represent any young girl in those countries. Clothes and looks were chosen specifically to be universal in the desired countries to make accepting the characters easier in any of the target countries. Meena is a nine-year-old girl who, through a series of cartoons, books, and radio programs, addresses gender inequality issues, dowries, child marriages, health issues, child labor, and other topics that are germane to the human rights goals of the Millennium Development Goals and Sustainable Development Goals (39).

**Figure 1:**
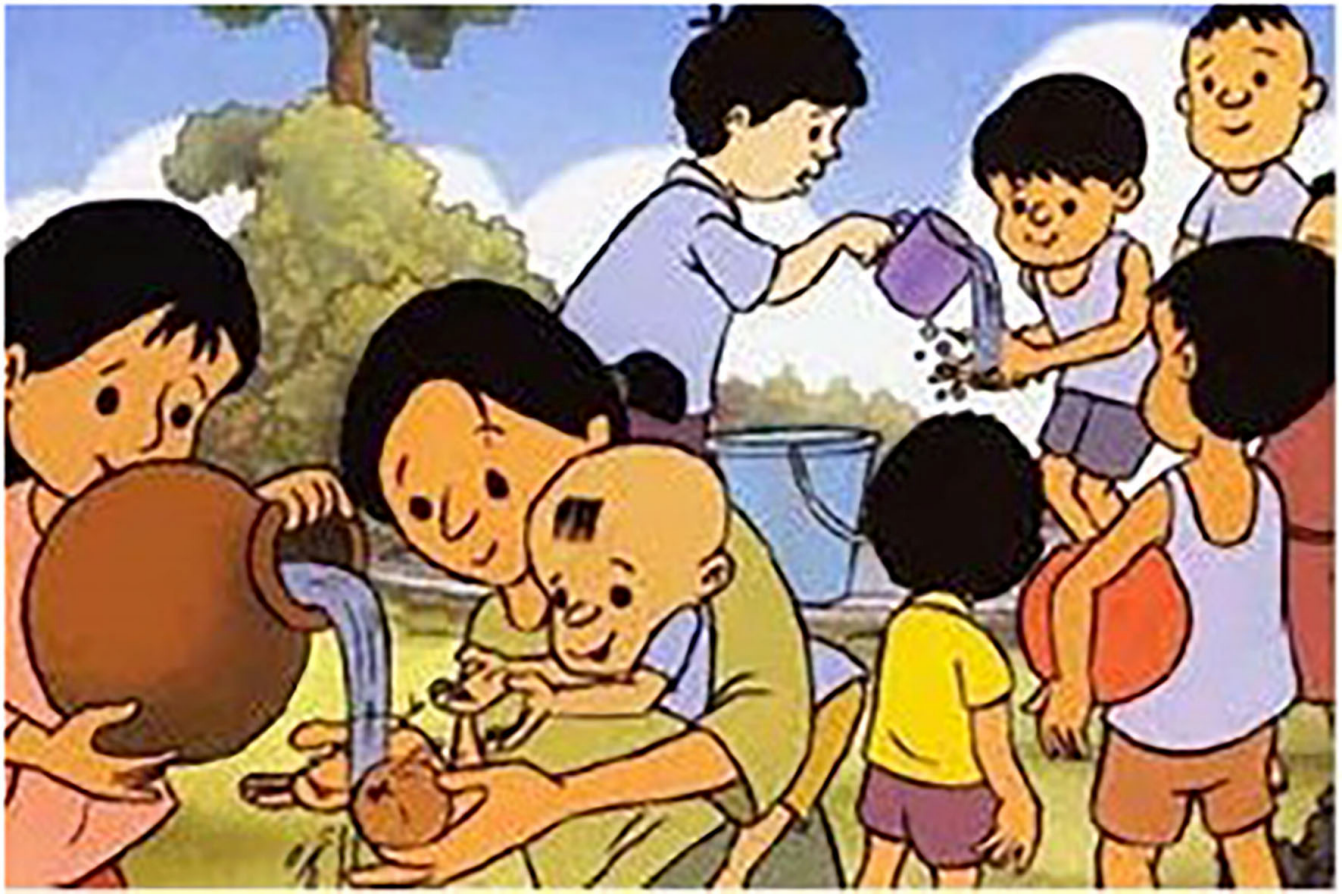
Still from Meena episode “Health in your Hands” (41).

Meena tackles issues that affect many girls in the target countries but also impact boys living in the same areas and gives an outlet for boys to become allies to the goals of Meena and by extension the girls in their communities. “She has also helped scores of boys dispel negative stereotypes surrounding girls and women, portraying equality in a way that appeals to younger generations,” says student Osama Rahman (40). The bulk of the 36 available episodes revolve around human rights issues, but there are at least seven episodes relating to public health, including hand washing shown in Figure 1, preventing worms, pediatric diarrheal disease, and others (42). A study in Nepal in 2002 found that 96% of children who interacted with Meena media (either through the tv show, radio programs, or books) reported making at least one behavioral change in their lives with the most mentioned change being handwashing (39).

Meena is still being watched by children in Southeast Asia and there is a Meena day recognized on September 24th in much of Southeast Asia. It was reported by the former communication director of UNICEF in Bangladesh that 97 % of children from urban areas and 81% of children from rural areas recognize Meena (42). Meena was considered the most effective strategy ever developed by UNICEF, with young adults in their late 20s continuing to remember the lessons that Meena taught them about handwashing and hygiene (39, 43). Recently a Meena game has been added to the Google Play store and has over 800,000 downloads (42).

### The Magic Glasses

The Magic Glasses is a 12-min cartoon developed for use in Hunan province China; however it could be and has been adopted in many provinces in China where STHs are endemic, featuring a school aged child named Xiaoxiong who is given special glasses that allow him to see larvae and eggs of soil transmitted helminths around his village. As he walks around the village and sees the places and people that are affected by worms, he decides to help the people in his village learn about the worms and how to avoid infection. With his help, the village becomes worm free but his parting advice to the villagers is to maintain their good hygiene or the worms will return. Chinese scientists were consulted to make the video culturally appropriate and more relatable to local students (44).

After some modifications based on feedback from the students involved in the pilot program, the positive response from children makes this an effective tool for educating school children how to avoid STH infections. In the intervention group of students who watched the video, there were 50% less children infected than in the control group who received a poster containing key information about STH. There was also an increase in children in the intervention group washing their hands after using the bathroom and before meals (45).

The children were asked beforehand what they knew about parasitic worms, causes and symptoms of those worms. This information was then used by a team of epidemiologists, social scientists, and civil engineers to create the cartoon. While this cartoon is in Chinese and designed to be used with Chinese students, the developers of this cartoon also created a second cartoon to be used in the Philippines (46). The Magic Glasses Philippines has its own animation, done in a style that Filipino children would respond to, and recorded in Tagalog as well as English. The data from the Philippines study are currently being analyzed and will lend some weight to the implications of the Magic Glasses being a successful health education tool that can be and has been adapted to many cultures. The video is available online (https://www.youtube.com/watch?v=7C-O5M3YnRE).

### Rama and the Worm

Indonesian traditional shadow puppet plays have been used to tell the stories of the Ramayana and Mahabharata, Indian epic stories about the Prince Rama and his journey. The Ramayana an integral part of the culture of Indonesia, particularly in certain populations such as the Balinese, Javanese and Sundanese people (47). It has been used to convey educational messages using the characters and storylines of the traditional shadow puppets, but in Rama and the Worm the researchers modified it to create a new narrative that still uses the familiar characters but puts them in a new situation that pertains to the education objective for the project. It was also shortened to 30 min in comparison to several hours to keep viewers engaged, although this was mentioned by at least one member in the focus group that the film was too short and should be at least 1 h long (48). Another significant change to the traditional show is the inclusion of Western instruments, combined with the traditional gamelan instrument to create a rock fusion soundtrack (47–49). In focus group discussions after the viewing, there were comments that it would benefit some villages, but was unnecessary for their own village citing that open defecation was not an issue in their community (48). Focus group members stated that the performance could be improved with the addition of a presentation by a health professional and materials, or a health message to be shown before the film (48). More than half of the people surveyed in the area lived in a house that did not have a latrine with a majority of those surveyed responding that they relied on public latrines. The main reasons stated for lack of latrine in the house is lack of funds to build or maintain, this has been noted and is being addressed by another study in the Javanese area of Indonesia (49). The “BALatrine”, an all-weather latrine designed and tested in Bali Indonesia, has been developed to be buildable with local materials and to be used during the wet season with a removable U-bend attachment or during the dry season as a dry pit latrine. This allows the use of the latrine in all seasons and the construction makes latrines more accessible to locals in rural and poor areas (50). Since ending open defecation is an important step in preventing transmission of STH and many other neglected tropical diseases, community engagement with the building of latrines and input into the designs used contribute to the acceptance and use of the BALatrines (50).

**Figure 2:**
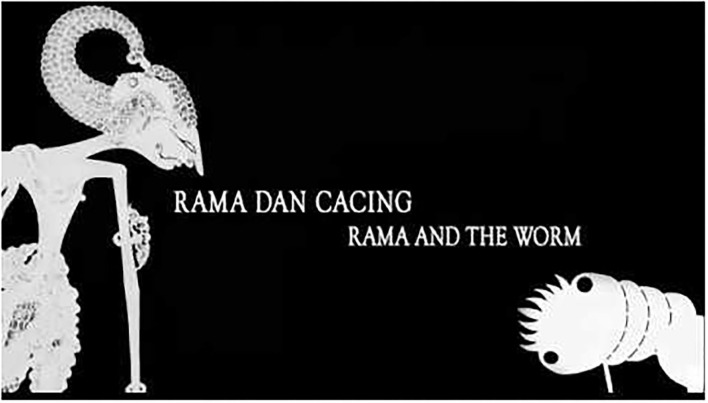
Still from Rama and the Worm shadow puppet play taken from Youtube.com (48).

While Rama and the Worm focuses on soil transmitted helminths, the insights gained from this could be easily translated into a culturally significant media education tool to combat other zoonotic diseases. The video is available online, cover art is shown in Figure 2 (https://www.youtube.com/watch?v=AafNmmMlrvQ).

### Snakes and Ladders

Snakes and Ladders is based off an ancient game that originated in India as a Hindu game to teach morality, it is also known as Chutes and Ladders in some countries as just a game with no morality lessons (51). Snakes and Ladders consists of numbered squares, some containing a head of a snake or the feet of a ladder. Players then roll a die to determine the number of squares they move forward, if they land on the feet of the ladder they climb up to the top of the ladder, if they land on the head of the snake they slide down to the bottom of the tail. The objective of the game is to be the first player to reach the 100th square. It has been used in several studies in Asia and Africa to evaluate health education intervention on schistosomiasis, *Taenia solium, Taenia saginata*. Games provide valuable learning opportunities, particularly when the game is entertaining, children often absorb more information from learning while playing (52, 53).

Schisto and Ladders was adapted from Snakes and Ladders in 2014 after students in Ogun State, Nigeria—West Africa, were refusing MDA with praziquantel. Out of 132 schools in the area, 78 schools refused MDA, with reasons including but not limited to concerns of side effects and risk of death with taking the medication. In the communities that have rejected MDA, each has a river that serves as the transmission site for schistosomiasis and have a lack of public water supplies or latrines. Most school children in the area visit the river everyday either to collect water for domestic activities or to wash or play in the water. In Schisto and Ladders, squares were decorated with a worm head containing messages with poor behavioral practices that contribute to the increased risk of schistosomiasis, and worm tails were added to squares that contained messages about the consequences of those risky behaviors (54). Ladders were located in squares with messages about good behavior practices and control messages.

Two versions were ultimately created, version 2 being an update and improvement on the previous version to increase compliance with MDA, using the ladder squares to convey positive messages about reducing the side effects of praziquantel, for example, eating before taking praziquantel to reduce the potential side effects (55).

As with most health education control, a pre and post activity assessment was performed to ascertain knowledge, attitudes, and practices (KAP) as well as focus group discussions with students and parents/caregivers. All of the children involved in the study had rejected the MDA praziquantel, either as their own decision or as directed by their parents. After playing the board game, 65% of students signed up for praziquantel treatment with their teachers, a large portion of the remaining students expressed interest in signing up but mentioned needing to check with parents before signing up. One important note to come out of the focus group discussion with parents was that they were not notified before the MDA, “Parents should be called and be informed of the MDA by the government”. If parents are not notified beforehand, they may give blanket statements to their children, as one student mentioned “ our parents said we should not take drugs in school” which ultimately undermines any control or intervention (55). Schisto and Ladders proved to be successful in increasing compliance with MDA programs and increasing positive behavior in students.

A second study using Snakes and Ladders was undertaken in Indonesia, as it was a game already familiar to children. Since the theme is adjacent to worms, it was a logical choice for modification to an informational game to educate students about *T. saginata, T. Solium* and taeniasis (51). The children's familiarity with the game also allows for quick uptake since the rules are mostly known to them already. The modifications of the game included 12 squares that if landed on, the students had to answer a question that the other player reads aloud. A correct answer moves the student forward while an incorrect answers sends them back.

This game was modified for a focally endemic area in Bali Indonesia, where consumption of raw beef or raw pork with blood is a traditional dish (lawar) (56). As Indonesia is a predominantly Muslim country consumption of pork is lower, however Bali is largely a Hindu area and pork consumption is higher there, although there is still significant beef consumption in Bali as well (56). This leads the area of Bali to be a prime candidate for broader taeniasis interventions of both *T. saginata* and *T. solium* and an ideal site to test the effectiveness of the educational game.

The effectiveness of this game as an interventional tool was ascertained by asking students to complete a pre- and post-game questionnaire when no previous educational interventions had been performed. Students were given a questionnaire with 10 fill in the blank questions to answer before playing the modified game for 30 min. After playing the game long enough for all students to hear the questions and answers for the 10 questions, they were given the post-game questionnaire containing the same questions as the pregame questionnaire. The number of correct answers increased by 18.5% between the pre and post-game questionnaires, showing that even a short amount of time interacting with the game could be beneficial to increasing knowledge about taeniasis (51).

## Africa

### Koko and the Lunettes Magiques

Koko et les lunettes Magiques is a cartoon that addresses common misunderstandings the children had while giving them the correct information instead. After researchers read about the study by Bieri in China with the Magic Glasses, they created an original cartoon based in Africa. Like the Magic glasses, the main character is an 8-year-old boy named Koko, who is given magic glasses by the local doctor that allow Koko to view his village as if it were under a microscope. This allows Koko to see the parasites infecting his village and himself, and how those worms are transmitted to humans. Koko then decides to inform the community of the risks of infection and how to prevent infections.

Overall the key messages from both cartoons are the same, and as with the Magic glasses, social scientists, civil engineers, and epidemiologists were consulted as well as focus groups with children to ascertain what they already know, where there are gaps and how to create a video that will be best accepted by children of the area. While this was designed for Cote d'Ivoire, it could be modified to reach children around Africa, especially if translations or sound dubbing was available to make the language accessible (57). By creating a new cartoon instead of translating the original Chinese version of the Magic Glasses, the researchers made the cartoon more relatable to their intended audience which will foster more interaction with the movie in the future.

**Figure 3:**
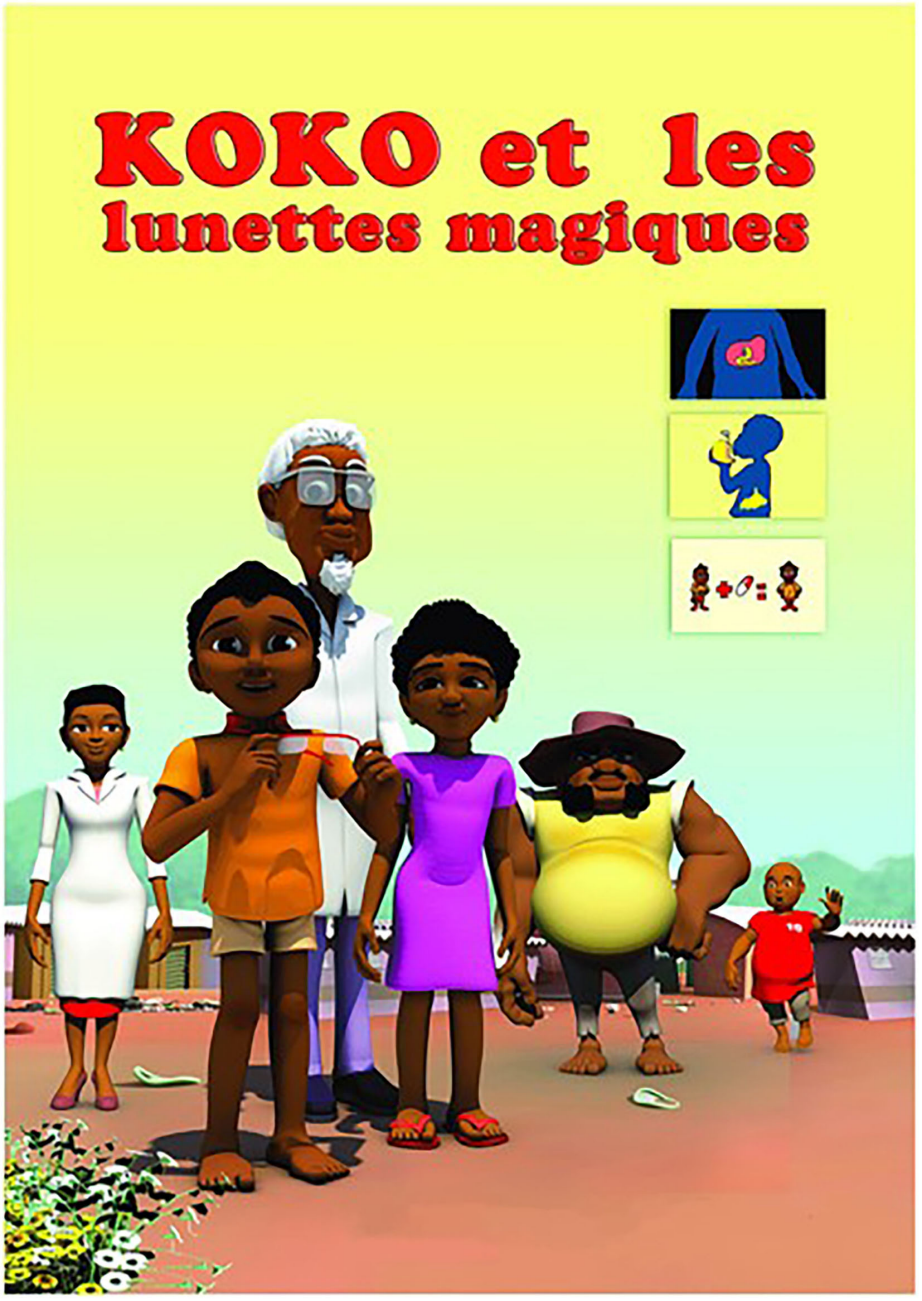
Koko et les lunettes magiques (57).

After watching the video, children were able to identify that hand washing and using latrines were good practices to avoid getting worms. Ninety-seven percent of participants indicated that hand washing after defecating and washing fruits before eating were important hygiene practices to avoid worms as well (57). The video is available to watch online, cover art is included in Figure 3 (https://www.youtube.com/watch?v=PCNLEK5Ityw).

### Juma Na Kichocho

Juma Na Kichocho is a comic book that was created to teach children in Zanzibar, Africa about schistosomiasis and malaria. The local children had health education in school on a regular basis about malaria, but no such education on schistosomiasis. The booklet was part of the Kick out Kichocho (Kiswahili for schistosomiasis) program initiated by the President of Zanzibar at the time. Juma is a young boy who learns about schistosomiasis /kichocho from his teachers and local health center staff. The comic book presents the information about symptoms, transmission and life cycles and control measures in a child friendly format. Before the comic books were disbursed, the students had a pre-exposure knowledge, attitudes, and practices (KAP) questionnaire, the books were handed out alongside a 30-min presentation by a health educator from the Helminth Control Laboratory Unguja. Over the next year, teachers were encouraged to include the booklets in their school health education, culminating in a post exposure questionnaire was given to assess KAP, the knowledge on the post questionnaire showed a decrease in malaria knowledge and no increase or decrease on schistosomiasis knowledge. This suggests that the comic book in question was not a successful health education intervention but does not indicate that comic books in general if presented a different way and in conjunction with other health education tools might not be successful (58, 59).

### The Vicious Worm

**Figure 4:**
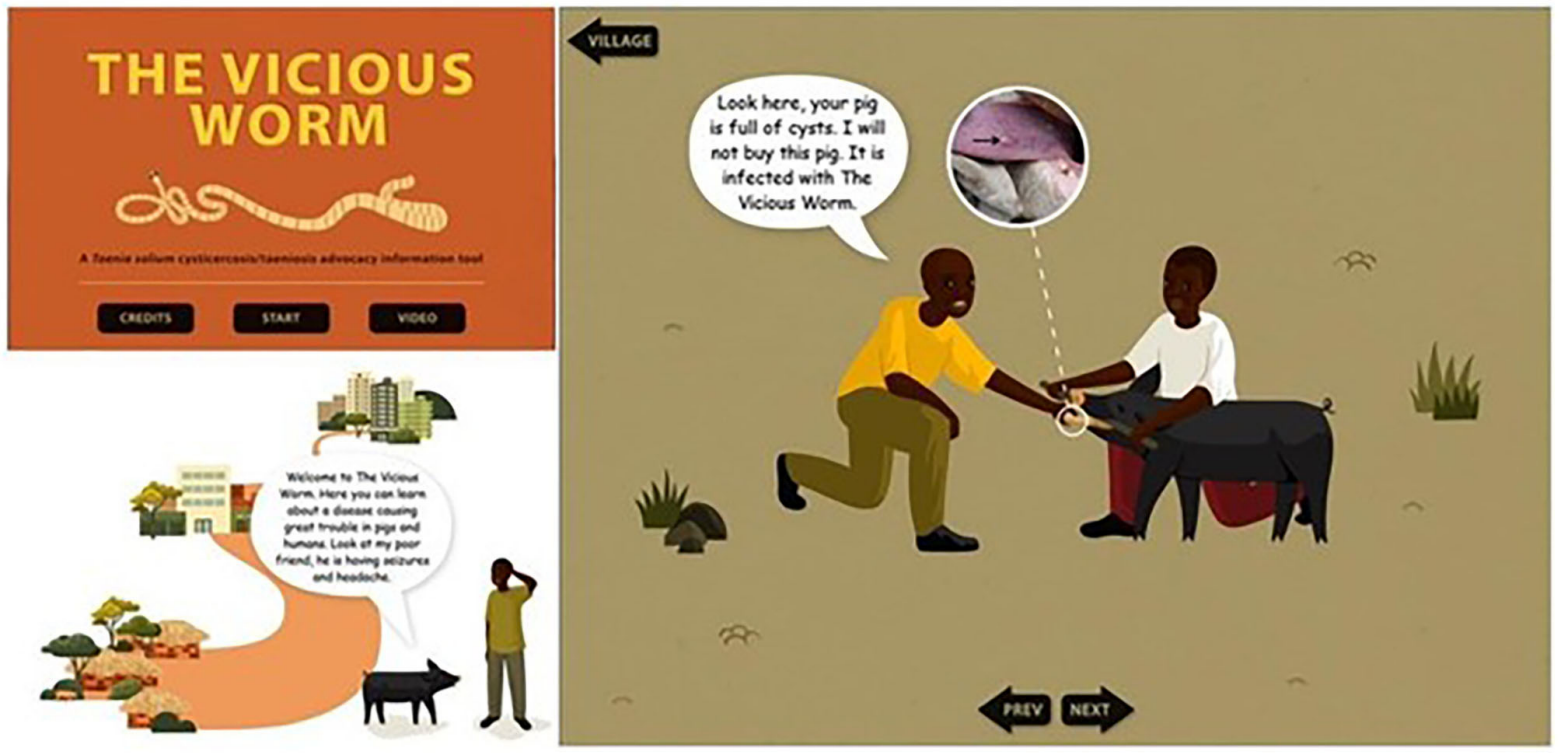
Screenshots of the Vicious Worm computer program (61).

The Vicious Worm is a computer program designed to teach about *Taenia Solium*, porcine tapeworm. There are three sections: (1) the village section was designed for lay people to interact with and uses more stories and pictures to disseminate the information, (2) the town section was targeted for medical practitioners, veterinarians, and meat inspectors, and (3) the city section was targeted for decision makers at policy levels (60). Each layer of the program provides information based on transmission, diagnosis, risk factors, prevention, and control of the disease. Within each are subsections on main topics; hand washing, buying, cooking, and consuming uninfected pork, pig raising, the life cycle of *T. Solium*, appropriate slaughtering, and meat inspection. The city section has a library with links for further research and reading on *T. solium* (60). A screenshot of the program is provided in Figure 4.

After the person has clicked on all the information sections, there is a schoolhouse where they can take a quiz based on what they should have learned. The original test of this program was given in Tanzania, East Africa to 79 subjects in the agriculture or health sectors and all of the participants scored 70% or higher on the pretest before the using the program, and all participants had a significant increase in knowledge when tested immediately after using the program for an hour and a half (62). A follow up test was done 2 weeks after using the program and all participants scored higher than their baseline scores, although some did not test as high as immediately following the program (62). There was a secondary follow up with the same participants that showed that a year after using the program, and the results were lower than the two-week post-test, but still higher than the original baseline. In addition, 82% of participants (50 people) had used the Vicious Worm program more than once either for personal learning or to engage with other people (63).

This program was given to school aged children in Zambia, East Africa for the initial study on effectiveness. Their knowledge was tested before using the program, after using the program and 1 year later in a follow up test. This study showed that knowledge retained after 1 year was less than right after using the program, but still more than pre-program knowledge (64). This education system is an example of One Health education as it discusses in detail not only how to prevent the worms from entering the human community, but also importance on rearing healthy pigs to stop transmission. While there are still improvements that could be made to include more environmental education, this program is the best example of One Health in an educational platform.

### Mobile Health (mHealth) Apps

Mobile Health (mHealth) apps and programs, apps that are used to monitor health, doctors' appointments and virtual doctors' visits, that are available on mobile phones will continue to be the most beneficial for many in LMIC because computers and tablets are still rarer and less people have access to those technologies (65). Mobile internet usage is the primary method of accessing the internet for people in LMICs, although smartphone use is lower than non-smartphone due to the cost. The average cost of a smartphone in LMICs fell from 44% of monthly income in 2018 to only 34% in 2019, making it more accessible for more people (66). This decrease in cost has been due to an increase in inexpensive devices available in Sub Saharan Africa and South Asia. Cost of the phones is the biggest barrier to mobile phone ownership in LMICs, although price is predicted to continue to fall and ownership is predicted to rise to 65% of connections in Africa being made with a smartphone by 2025 (66). Smartphone usage is higher among youths and those with higher education levels, as literacy is also a barrier for some mobile users in LMIC (67). Additional benefits to mobile/computer/tablet based educational programs is that lessons or programs can be modified for weaker students by including reviews for stronger students with advanced modules. They can also be reused and redistributed with no wear and tear as opposed to poster or pamphlets which are consumable goods. Computer programs, apps for phones and tablets, and web based media enable learning at any time in any place, a system which can benefit workers who would not be able to access these programs during working hours (34).

mHealth apps can be informational in design like apps for maternal health such as the Text4Baby app or Mobile Midwife. These provide mothers to be with information about having a healthy pregnancy, how to avoid getting malaria while pregnant, and tips on delivery and childbirth (68). There are numerous mHealth apps that can give the user information about their specific disease or provide symptom tracking.

A benefit of the mHealth apps is that with them, health officials are able to disburse information needed for training or medical materials to community health workers. For example, in Nigeria during the Ebola outbreak in 2014 the Adandach Group created a mobile tutorial for health care workers explaining the causes of Ebola, ways to diagnose Ebola, how it spreads, and how to treat it in the field (68). This knowledge helped healthcare workers who had been unaware of how the disease spreads better protect themselves and patients who came into their clinics (68). During the Covid-19 pandemic, several apps were developed to monitor potential Covid symptoms as well as contact tracing and notifications, this could be used for future outbreaks of Ebola or other pandemics. These apps have less practical use for most NZDs but could be a potential source of reporting cases.

In addition to this program there are a dozen similar programs in other countries using this technology to help healthcare workers perform their jobs with up-to-date materials or communicate with patients in hard-to-reach areas. There are also mHealth apps which remind patients about future appointments or maintaining their therapeutic medicine schedules, this frees up healthcare providers who would normally be tasked with those reminders and reduces nonattendance at appointments by as much as 40% (68).

There is a greater potential for mhealth apps aimed at NZDs, including giving people in remote areas access to emergency medical advice that would normally not be an option given the remoteness of their location. Medical professionals can be texted, and a response received in under 3 min, as is the case with the Clinton Health Access Initiative's cooperative venture with IT group HP, giving remote and poorer areas a lifeline in an emergency (65). This can give a family time to perform triage on members and buy them time to get to medical professionals if the situation warrants, potentially saving the family time and money if it is a situation that can be managed at home.

During the Covid-19 pandemic, there have been multiple new mHealth apps related to contact tracing, symptoms tracking, and new advancements in Telehealth virtual communications. While these were created to help end the spread of Covid, these programs can have long reaching benefits to those in rural communities to have access to a telehealth appointment with doctors, eliminating the need and cost of expensive travel to see a doctor. Seeing a doctor via a video chat platform available on most mobiles would enable more people to see a doctor before their symptoms became too severe to be helped or while still in the early stages making it easier and less costly to treat (69, 70). Symptom and contact tracing apps can also be used to monitor infectious diseases to prevent the spread, such as Ebola, Nipah Virus, and other NZDs (71).

### Conclusions

It is clear that several health education interventions are more successful than others, with a few having a lasting effect on the viewer or user. There are many opportunities to modify and expand educational programs to be more effective in more countries. Efforts like the Meena Communication Initiative can have long lasting impacts on children, with adults today remembering the lessons they learned. The MCI was well funded by UNICEF as well as several governments and is by far the most funded of any of the interventions listed in this paper at an estimated $7,837,000 USD. Perhaps then it is no surprise that it appears the most effective intervention of its kind. An underlying theme through all of the education tools included is a lack of funding to embed them in the intended communities, as is often the case with neglected diseases, whether tropical or zoonotic. Another important factor in health education is the balance between education and entertainment. Too much information with too little entertainment is quick to bore; too much entertainment and the health message is lost in the drama (72). The Meena episodes were able to achieve that balance with adventures that taught while being entertaining.

One potential success of the MCI is that it reached girls and women who brought those messages forward for their children, especially their daughters.

One drawback to most of the educational health studies is that they were performed in the early 2000s, and there have been few in recent years. A more in-depth assessment of the effectiveness of health education is needed as well as definitive data on post activity behavioral changes both in students and in their communities. Many of the studies on the effectiveness of health education are related to maternal health. Far fewer are on neglected tropical diseases focused on children. There is a great potential with the advancements in children's programming to incorporate more health education messages into already popular cartoons and media.

Since the bulk of the studies are from the early 2000s, they don't reflect the advances in technology and increase in availability of internet and mobile services as well as the increased ease of use of technology amongst young people. The more widespread availability of internet creates opportunities for edutainment media to be seen by more children and families with the potential for greater possible impact. Smartphones and internet have become ubiquitous in developed countries and their use continues to rise in LMIC as cost of ownership falls steadily (73). There are many good educational apps for mobile devices and computer-based games, including several that have come along since the COVID-19 outbreak created the need for online education for most schools around the world. As a result of lock downs and school closures due to COVID-19, the need for virtual education tools was seen as a temporary measure, however as the pandemic continues into the second and potentially into the third year, virtual classrooms and education apps are still a necessity.

Further studies are needed to determine the efficacy of these new learning tools and how they can be modified to help with community health education programs for a variety of diseases, including neglected tropical and zoonotic diseases in LMICs. There is more that can be done with online classrooms to ensure education equity in LMICs as well as continuing education for children who are limited by illnesses from attending school. Health education is an underused tool, used effectively it can reduce the number of cases of NZDs and create lasting impressions on children. New educational programs should be developed to address the changes in the environment and encourage community involvement in sanitation practices.

## Author Contributions

CB was responsible for conception, assimilation of works, and drafting of the paper. JF and SW were involved in conception and editing the manuscript. All authors contributed to the article and approved the submitted version.

## Funding

The authors acknowledge support from Zhejiang University (SW, CB) and acknowledge research support from Tackling Infections to Benefit Africa, National Institute of Health Research, using Official Development Assistance (ODA) funding 16/136/33 (SW, CB). The funders had no role in the study design, data collection and analysis, decision to publish or in preparation of the manuscript.

## Conflict of Interest

The authors declare that the research was conducted in the absence of any commercial or financial relationships that could be construed as a potential conflict of interest.

## Publisher's Note

All claims expressed in this article are solely those of the authors and do not necessarily represent those of their affiliated organizations, or those of the publisher, the editors and the reviewers. Any product that may be evaluated in this article, or claim that may be made by its manufacturer, is not guaranteed or endorsed by the publisher.
